# Breast cancer screening patterns in Jamaican women: review of the largest national mammography clinic

**DOI:** 10.1186/s12905-021-01295-4

**Published:** 2021-04-23

**Authors:** Sheray N. Chin, Derria Cornwall, Derek I. Mitchell, Michael E. McFarlane, Joseph M. Plummer

**Affiliations:** 1 Department of Pathology (Division of Haematology & Oncology), Faculty of Medical Sciences, University of the West Indies, Mona, Jamaica; 2 Department of Surgery, Radiology, Anaesthesia and Intensive Care, Faculty of Medical Sciences, University of the West Indies, Mona, Jamaica

**Keywords:** Breast cancer, Screening, Mammogram

## Abstract

**Background:**

Breast cancer is the leading cause of cancer and cancer related deaths in Jamaican women. In Jamaica, women often present with advanced stages of breast cancer, despite the availability of screening mammography for early detection. The utilization of screening mammography for early breast cancer diagnosis seems to be limited, and this study investigated the national patterns of mammographic screening and the impact of mammography on the diagnosis of breast cancer in Jamaica.

**Methods:**

A retrospective analysis of the records of the largest mammography clinic in Jamaica was done for the period January 2011 to December 2016. Descriptive statistics was performed on relevant patient characteristics with calculation of rates and proportions; cross-tabulations were utilized to assess relationship of covariates being studied on the outcomes of interest. Results are reported in aggregate form with no identifiable patient data.

**Results:**

48,203 mammograms were performed during the study period. 574 women (1.2%) had mammograms suspicious for breast cancer with median age of 57 years (range 30–95 years); 35% were under the age of 50. 4 women with suspicious findings had undergone ‘screening mammography’, with the remaining having ‘diagnostic mammography’. 38% reported previous mammograms, with a mean interval of 8 years between previous normal mammogram and mammogram suspicious for breast cancer. Median age at first screening mammogram was 51 years (range 41–77).

**Conclusion:**

Breast cancer screening mammography is underutilized in Jamaica. An organized national breast cancer screening programme is recommended to improve adherence to international breast cancer screening guidelines.

## Background

Breast cancer is the leading cancer affecting women and the leading cause of cancer related deaths in females in Jamaica [1, 2]. Late presentation of breast cancer is prevalent, with many women presenting with large tumors (median tumor size 3.5 cm, ranging from 0.4 cm up to 13 cm), with histologically confirmed axillary lymph node involvement reported to be as high as 56–76% [3, 4]. This makes locally advanced breast cancer a common presentation in Jamaica. Low utilization of breast cancer screening mammography in the population is a possible contributing factor to this.

Despite the high burden of breast cancer and the widely accepted benefits of screening mammography, opportunistic screening predominates and relatively few women in Jamaica have age-appropriate regular screening mammograms. It has been reported that less than five per cent of Jamaican women eligible for mammographic screening actually have mammograms [5], and local studies have explored the deterrents to mammographic screening [6]. This study assesses the impact of screening mammography on the diagnosis of breast cancer in this population.

Breast cancer in Jamaican women is diagnosed at a median age of 52 years [4], which is an average of 10 years younger than in the USA [7]. The diagnosis of breast cancer in a relatively young population has the inherent issue of decreased sensitivity of mammography in detecting small cancers due to increased breast density [8]. One local study showed that as many as 17% of women with breast cancer were under the age of 40 years [9], with a peak of 53% in the 41 to 60 year age group; this is in contrast to the age distribution of breast cancer cases in the US, where fewer than 5 percent of women diagnosed with breast cancer are younger than 40, and the highest rates are seen in the over age 70 years demographic [10].

The Jamaica Cancer Society (JCS) is a non-profit, non-governmental organization that has been offering breast cancer screening for over three decades and has the largest mammography clinic in the country, with up to 9000 mammograms done annually. The main screening site is located at the JCS office in the Kingston metropolis, with ancillary mobile screening services provided by a mobile mammography unit which serves urban as well as rural areas. The JCS breast cancer screening programme has had a collaborative relationship with the University of the West Indies (UWI) and the University Hospital of the West Indies (UHWI) for many years, with consultant radiologists and surgeons from the UHWI/UWI providing radiological and clinical services. Once a mammogram is deemed “suspicious for breast cancer”, the patient is contacted, and a note made to the referring doctor recommending referral to Surgery for management, which usually involves further investigation of the suspicious mass with biopsy. Self-referred clients are offered referral to the JCS Breast Clinic, with some opting to pursue private care. Women with a confirmed diagnosis of breast cancer are referred to the JCS counseling service &/or their support group, Jamaica Reach to Recovery.

In this study we explored the patterns of breast cancer screening at the JCS between 2011 and 2016. The overall aim of this study was to establish the impact of breast cancer screening mammography on the diagnosis of breast cancer in Jamaica.

## Methods

A retrospective analysis of the JCS mammography records from January 2011 to December 2016 was done. Demographic and clinical data were recorded for all women.

Data was collected from the archives of the JCS. A paper-based record is maintained for each client who presents to the  JCS  for mammography. Demographic data (age, parish of residence), as well as clinical data (presenting symptoms/signs, menstrual history, parity, prior mammogram history, personal/family history of breast cancer) are recorded on enrolment. Mammograms are reviewed by reporting radiologist, and these reports (radiologist’s diagnosis and recommendations) are also recorded in the patient record. These were reviewed as part of this study, with manual reviews performed by two reviewers (D.O., S.C.).

Women who presented with a symptomatic breast complaint were considered to have undergone ‘diagnostic mammogram’, while mammograms for patients who were asymptomatic at presentation were deemed ‘screening mammograms’. For those with a suspicious mammogram (as per reporting radiologist), we ascertained from the clinical records whether or not they had breast symptoms/ signs. We considered those who were asymptomatic at the time of mammogram and diagnosed with breast cancer to have “screen-detected breast cancer”.

Descriptive statistics was performed on relevant patient characteristics. Quantitative variables such as age at presentation were grouped into age groups, using decade intervals for the usual age groups eligible for screening mammography (40–49; 50–59; 60–69; 70–79 years). For the younger ages, these were grouped as under 35 years and 35–39 years and the older age group as 80 years and over.

Rates and proportions were calculated and cross-tabulations were utilized to assess relationship of covariates being studied on the outcomes of interest. Results are reported in aggregate form with no identifiable patient data.

## Results

**Fig. 1:**
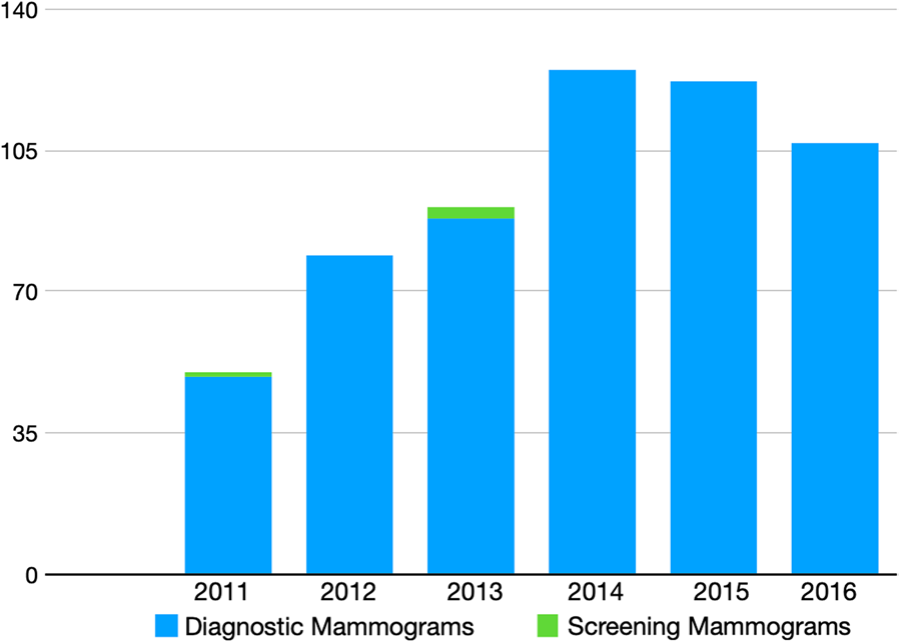
Diagnostic versus screening mammograms, for 571 mammograms reported as ‘suspicious for breast cancer’ at the JCS, 2011–2016

During the 6-year study period, 48,203 mammograms were performed at the JCS, with a mean of 8033 mammograms annually. A total of 574 women had mammograms reported as suspicious for breast cancer (mean 1.2%) (Fig. 1). 51% of these women were from the Kingston and St. Andrew region, in keeping with the geographic location of the JCS screening clinic, however all parishes were represented. Seventy percent of women who presented for mammography were in the 40–59 years age group, with 2% younger than 40 years of age (Table 1). Almost half of those who presented for breast cancer screening had no prior screening mammogram (45%) and for those women utilizing the mobile facility, 61% had never had a mammogram.

**Table 1:** Age distribution of 48,203 women who had mammograms at the JCS, during the period 2011–2016

Age range (years)	% of women (no)
Under 35	0.4 (173)
35–39	1.5 (705)
40–49	35.1 (16,941)
50–59	34.9 (16,842)
60–69	20.5 (9884)
70–79	6.4 (3092)
80 and over	1.4 (667)

Radiologist’s diagnosis and detailed recommendations after mammogram review were available for mammograms performed during 2014–2016 (25,529 mammograms). Approximately 19% of imaged women were recommended to have breast ultrasound for further imaging and 7% were noted by the reporting radiologist as having “dense breasts”. A radiologic diagnosis of benign breast disease (fibroadenoma, fibrocystic disease, duct ectasia) was made in 16% of all imaged women, with the majority of these (76%) being fibrocystic disease (Table 2).

**Table 2:** Radiologist’s diagnosis and recommendations after mammogram review, available for 25,529 mammograms performed at the JCS, 2014–2016

Radiological diagnosis/recommendations	% of 25,529 mammograms (no)
No abnormality or other comment	50.9 (13, 003)
Any abnormality	49.5 (12,526)
Ultrasound recommended	18.7 (4699)
Dense breasts	6.7 (1716)
Benign breast disease	16.2 (4132)
Fibrocystic breast disease	11.9 (3135)
Duct ectasia	2.7 (635)
Fibroadenoma	1.5 (362)

### Mammograms suspicious for breast cancer

The median age of the 574 women with mammograms suspicious for breast cancer was 57 years, with a range of 30–95 years; 35% were under the age of 50 years, and 4% were younger than 40 years (Table 3).

**Table 3:** Age distribution of 571 women who had mammograms suspicious for breast cancer at the JCS, 2011–2016

Age range (years)	% of 571 women (no)
Under 35	0.7 (4)
35–39	3.7 (21)
40–49	30.1 (172)
50–59	28.5 (163)
60–69	18.0 (103)
70–79	13.7 (78)
80 and over	5.3 (30)

Only 4 of these women were recorded as not having had any signs/symptoms of breast cancer (screen-detected), and they had the following characteristics: (1) 55 years of age, family history of breast cancer (paternal grandmother, age at diagnosis unknown), (2) 60 years, 2 previous normal screening mammograms (dates not recorded), no family history of breast cancer, (3) 53 years, normal screening mammogram at age 49 years, family history of breast cancer (aunt, age at diagnosis unknown) and (4) 73 years with a personal history of breast cancer (previous contralateral mastectomy).

We found that 38% of women with a suspicious mammogram reported having had a mammogram in the past, but it was difficult to ascertain details of previous history of screening mammogram as this information was not routinely recorded for most of the study period. However, for the 2-year period for which records on previous mammograms were available, the median age at first screening mammogram was 50 years (range 37–77), and women reported having a mean of 3.8 mammograms performed over their lifetimes. The mean interval between previous normal mammogram and suspicious mammogram was 8 years.

### Breast cancer risk factors

A family history of breast cancer was noted for 23.7% of women with a suspicious mammogram, with 10.7% of women reporting an affected first degree relative. Median parity was 3 (range 0–15), with only 1 nulliparous woman in the group; median age at first childbirth was 19 years of age (range 13–42 years). Median ages at menarche and menopause were 13 years (range 11–16), and 50 years (range 40–56) respectively. Five percent of women with suspicious mammograms had a personal breast cancer history, with mammogram as part of their surveillance.

## Discussion

We found that very few women with suspicious findings on mammogram had actually undergone true screening mammograms. Most women had suspicious clinical breast findings that prompted diagnostic imaging as part of their work-up for a diagnosis of breast cancer. This is supported by previous clinicopathologic studies of breast cancer in Jamaica which showed that many women present with clinically significant disease, with large palpable tumors possibly with a diagnosis of locally advanced breast cancer [3, 4].

Interestingly, review of traditional breast cancer risk factors (e.g. nulliparity, late age at first parity) in women with a suspicious mammogram showed that these were not commonly reported. This is in keeping with previous reports showing a low prevalence of traditional breast cancer risk factors in Jamaican women diagnosed with breast cancer [11]. Identification of Jamaican women at an unusually high risk for the development of breast cancer based on the presence of traditional risk factors is therefore challenging. Further to this, genetic risk factors such as the presence of established predisposition genes for hereditary breast and ovarian cancer (e.g. BRCA1, BRCA2) have not been found to be prevalent in Jamaican women, compared to other Caribbean countries [12]. Research in this area is ongoing.

There is concern about the sensitivity of mammography in the presence of dense breasts [8], which is known to be an independent risk factor for breast cancer development [13]; dense breasts was reported for 7% of women in this study. Ancillary imaging with breast ultrasonography may be indicated for these women, and we noted that 19% of women were recommended to have same. Ultrasound services at the point of service may therefore help to increase detection rates.

We found that the mean interval between previous normal mammogram and mammogram suspicious for breast cancer was 8 years. This long interval between screening is especially of concern given the high prevalence of aggressive biology breast cancer in Jamaican women, with the potential of rapid tumour progression. We believe that screening will be more beneficial if intervals between screens are shorter (whether annual or biennial as per published recommendations [14]). In this study, many women were having their first mammogram, although most were 50 years of age or older (63%), and almost one-third were 60 and over, indicating late initiation of breast cancer screening, which is concerning given the relatively earlier age of breast cancer diagnosis in Jamaica.

Limitations of this study assessing the impact of screening mammography on breast cancer diagnosis in Jamaica are acknowledged and include that this was a retrospective study of one unit that offers mammography. However, the mammographic clinic at the JCS was felt to be representative as it is the largest national screening clinic and includes women from all parishes and health regions in the country. While clinical records were available for all women, another limitation is that there were no data available on tumor characteristics (for any of the women), as the Jamaica Cancer Society did not record clinical stage (tumor size/ nodal status). For women who subsequently went on to have surgery, there were no copies of histology reports to determine pathological staging. Recording of clinical and pathological staging would have been useful to support our premise that screening would lead to earlier breast cancer diagnosis/ stage migration. We have recommended that these be recorded as part of the clinical records, as this would be very useful data for future studies.

The performance indicators required for comparison to international benchmarks were reviewed. Unfortunately the data available in this study/ recorded in the mammogram records do not include the details required to use this metric, and this is an acknowledged limitation. As we strive to develop an organized national screening program, we will use this or similar international benchmark and record data necessary to compare the performance indicators to acceptable international standards.

## Conclusions

Breast cancer screening mammography is underutilized in Jamaica. Education and outreach are recommended to educate health care workers and the public about breast awareness, breast cancer risk in women, and screening mammography. An organized national breast cancer screening programme is recommended to improve adherence to international breast cancer screening guidelines, specifically recommendations for age of initiation, and screening intervals, in order to increase early detection and reduce the burden of advanced presentations of breast cancer.

## Data Availability

The data that support the findings of this study are available from the Jamaica Cancer Society, but restrictions apply to the availability of these data, which were used under license for the current study, and so are not publicly available. Data are however available from the authors upon reasonable request and with permission of the Jamaica Cancer Society.

## Abbreviations

JCS: Jamaica Cancer Society
UWI: University of the West Indies
UHWI: University Hospital of the West Indies

## Declarations

### Authors' information

SC is a medical oncologist with a special interest in reducing the burden of locally advanced breast cancer and reducing disparity in breast cancer outcomes. DC is a radiologist specializing in breast imaging, including mammography for early breast cancer detection. JP, MM and DM are general surgeons with expertise in breast cancer management. All authors are advocates for breast cancer screening and early detection.

## References

[1] Gibson TN, Hanchard B, Waugh N, McNaughton D (2010). Age-specific incidence of cancer in Kingston and St Andrew, Jamaica, 2003–2007. West Indian Med J.

[2] Blake G, Hanchard B, Mitchell K, Simpson D, Waugh N, Wolff C (2002). Jamaica cancer mortality statistics, 1999. West Indian Med J.

[3] Shirley SE, Sinclair PA, Stennett MA, Codrington G, Bhatt R, Escoffery CT (2010). The pathology of breast cancer in Jamaica: The National Public Health Laboratory Study. West Indian Med J.

[4] Chin SN, Green C, Strachan GG, Wharfe G (2014). Clinico-pathological characteristics of breast cancer in Jamaica. Asian Pac J Cancer Prev.

[5] Soares D, Kirlew K, Johnson P, Reid M (2007). Mammographic referral patterns for two breast imaging units in Jamaica. West Indian Med J.

[6] Soares D, Walters N, Frankson M, Kirlew K, Reid M (2009). Sociocultural deterrents to mammographic screening in Jamaica. West Indian Med J.

[7] Howlader N, Noone AM, Krapcho M, et al. (editors). SEER Cancer Statistics Review, 1975–2014. Table 1.12. National Cancer Institute. Bethesda, MD, http://seer.cancer.gov/csr/1975_2014/, 2017.

[8] Checka C, Chun J, Schnabel F, Lee J, Toth H (2012). The relationship of mammographic density and age: implications for breast cancer screening. Am J Roentgenol.

[9] Chin SN, Green C, Gordon-Strachan G, Wharfe G (2016). An audit of haematology/oncology clinic services at an urban academic hospital in Jamaica. West Indian Med J.

[10] American Cancer Society. Breast Cancer Facts and Figures 2017–2018. Atlanta, GA: American Cancer Society; 2017.

[11] Brady-West DC, Graham SA (2000). Prevalence of risk factors in breast cancer patients at the University Hospital of the West Indies. West Indian Med J.

[12] Lerner-Ellis J, Donenberg T, Ahmed H, George S, Wharfe G, Chin SN (2017). A high frequency of PALB2 mutations in Jamaican patients with breast cancer. Breast Cancer Res Treat.

[13] Boyd NF, Guo H, Martin LJ, Sun L, Stone J, Fishell E (2007). Mammographic density and the risk and detection of breast cancer. N Engl J Med.

[14] Siu AL, on behalf of the USPSTF. Screening for breast cancer: U.S. preventive services task force recommendation statement. Ann Intern Med. 2016;164(4):279–96. 10.7326/M15-2886 26757170

